# Emotional AI in education and toys: Investigating moral risk awareness in the acceptance of AI technologies from a cross-sectional survey of the Japanese population

**DOI:** 10.1016/j.heliyon.2024.e36251

**Published:** 2024-08-13

**Authors:** Manh-Tung Ho, Peter Mantello, Quan-Hoang Vuong

**Affiliations:** a Centre for Interdisciplinary Social Research, Phenikaa University, Ha Dong, Hanoi, 100803, Viet Nam; b Institute of Philosophy, Vietnam Academy of Social Sciences, Hanoi, 100000, Viet Nam; c Ritsumeikan Asia Pacific University, Beppu, Oita, 874-8577, Japan

**Keywords:** Emotional AI, Smart toys, EdTech, Technological acceptance model, Moral foundation theory

## Abstract

Emotional artificial intelligence (AI), i.e., affective computing technologies, is rapidly reshaping the education of young minds worldwide. In Japan, government and commercial stakeholders are promulgating emotional AI not only as a neoliberal, cost-saving benefit but also as a heuristic that can improve the learning experience at home and in the classroom. Nevertheless, critics warn of a myriad of risks and harms posed by the technology such as privacy violation, unresolved deeper cultural and systemic issues, *machinic parentalism* as well as the danger of imposing attitudinal conformity. This study brings together the Technological Acceptance Model and Moral Foundation Theory to examine the cultural construal of risks and rewards regarding the application of emotional AI technologies. It explores Japanese citizens’ perceptions of emotional AI in education and children's toys via analysis of a final sample of 2000 Japanese respondents with five age groups (20s–60s) and two sexes equally represented. The linear regression models for determinants of attitude toward emotional AI in education and in toys account for 44 % and 38 % variation in the data, respectively. The analyses reveal a significant negative correlation between attitudes toward emotional AI in both schools and toys and concerns about privacy violations or the dystopian nature of constantly monitoring of children and students’ emotions with AI (Education: β_DystopianConcern_ = − .094***; Toys: β_PrivacyConcern_ = − .199***). However, worries about autonomy and bias show mixed results, which hints at certain cultural nuances of values in a Japanese context and how new the technologies are. Concurring with the empirical literature on the Moral Foundation Theory, the chi-square (Χ^2^) test shows Japanese female respondents express more fear regarding the potential harms of emotional AI technologies for the youth's privacy, autonomy, data misuse, and fairness (p < 0.001). The policy implications of these results and insights on the impacts of emotional AI for the future of human-machine interaction are also provided.

## 1 Introduction

Affective computing, more widely known by its commercial name, emotional AI, is an interdisciplinary field combining advances in computational modeling, natural language processing, voice recognition, heart rate, respiration rate, galvanic skin response, gait, and facial analysis to endow digital devices with the ability to read, classify, and respond to human emotions. Commonly mistaken for sentient AI, emotional AI refers to intelligent machines and software that can effectively monitor, read, reliably recognize and evaluate emotions in humans [1]. The field dates back to Rosalind Picard's foundational research in 1995 [2]. The present-day development of emotional AI has been accelerated by continuing advances in big data, data mining techniques, deep learning algorithms, and a return of knowledge-based approaches to AI first developed in the 1990s and early 2000s. Leading emotional AI scholars in the field, have already found commercial success with these technologies. For example, Picard's *Affectiva* and *Empatica* offer a commercialized range of affect tools that can detect real-time emotions such as frustration, stress, happiness, etc. via psycho-physical (biometric) data captured through wearable devices [3]. Bjoern Schuller, from Imperial College London, co-founded *audEERING*, which manufactures emotion-sensing devices for audio media, and Erik Cambria, Nanyang University of Technology, co-founded *SenticNet* which applies state-of-the-art sentiment analysis software for marketing. These companies are leading a global industry worth over 21 billion US dollars which is expected to double by 2025 [4] (see Table 8).

Though a nascent industry, emotion-recognition devices and software are quickly becoming de facto tools in counter-terrorism [5], law enforcement [6], healthcare [7], advertising [8] video-gaming [9]; automobiles [10]; and the workplace [11,12]. Given its ability to detect distraction, attentiveness, and engagement, emotional AI is also garnering a reputation as a preeminent pedological aid for augmenting the development of young minds. As a result, emotion-sensing technologies are not simply being sold as an effective cost and timing-saving teaching aid to educational institutions but also as a children's toy, delivering personalized learning and gameplay to enhanced interventions for children struggling emotionally such as with autism [13] or loneliness [14]. A good example is *4 Little Trees*, an AI system developed in Hong Kong that gauges not simply ‘attention’ and ‘motivation’ levels of young learners but goes so far as to forecast their grades [15].

The prevailing logic behind enhancing edtech with emotional AI is an emerging belief that the learning experience of youth should not simply focus on their academic skills but also on the development of their emotional and social intelligence. This is because these soft skills are vital to decision-making [16], social relations [1], and ultimately, learning [17]. However, a growing number of scholars point to the innate dangers and ethical risks [18,19] of such a techno-deterministic approach to pedagogy. For example, Williamson (2021) notes the unproven track record of emotion-sensing devices, their lack of accuracy in reading emotions, and the lack of inclusiveness of the training data in emotional AI edtech [17]. Following Williamson, McStay (2020) suggests that the panopticon nature of the technology may increase feelings of inhibition and excessive self-consciousness in students [20]. In a study of the impact of conversational AI on loneliness, Sullivan et al. (2020) observe that human-machine relations are likely to be stronger when the AI is perceived to be more human-like. Yet they also note that the same perceived level of humanness in the AI may lead to the uncanny valley phenomenon where a child's construal of the anthropomorphism turns creepy [14].

Moreover, McStay and Rosner [21] call attention to the ‘generational unfairness’ with smart toys where adults not only make decisions for children about which toys they can play with but also lack technical literacy to appropriately assess the potential risks and harms facilitated by these smart toys. These potential risks not only include physical risks of tripping, battery heating, or malfunction but also psychological and environmental risks of erosion of children's agency and autonomy, privacy violation, unsustainability, etc. [18,19]. Compounding this problem is the potential for what we call, ‘*machinic parentalism*’, the situation where adults use these toys and digital devices to occupy a child's attention but also compensate for physical interaction with them. This trend can already be witnessed in a growing number of parents relying on mobile games to distract their children while traveling or when they are engaged in domestic chores or remote work [22,23].

This last point is particularly salient since, in all likelihood, this new genus of toys will not only become smarter but also more human-like with the latest advent in generative AI [24].

Regardless of these issues, emerging smart technologies such as emotional AI or generative AI are being integrated with high volume, velocity, and virality into all aspects of society either by their innate commercial viability or by government's will. For example, the Japanese government has launched a 3.12 billion (USD) initiative to address what they believe is an ongoing deficit in digital learning experience in nationwide education [25]. In the next section, we describe the current trends and narratives of smart technology use in the Japanese education system. Although much has been written about the application of AI and EdTech in the West [[26], [27], [28]] the *social and ethical considerations* of emotional AI Japanese context are overlooked and understudied.

### 1.1 The context of AI and emotional AI in Japanese educational setting

Augmenting traditional teaching approaches and youth learning experiences in Japan with AI is premised on solving two major issues. First, is connected to the nation's declining population and lack of fresh sources of human labor to replenish teaching staff in the educational system [29]. This problem is compounded by the growing stigma attached to the teaching profession. Japanese schoolteachers are known to be massively overworked without added compensation [30]. For younger generations entering the workforce, the prospect of becoming a primary or secondary school teacher does not carry the same social esteem as it once did. As a result, they are choosing other careers which, in turn, exacerbates the ongoing human resource drain on Japanese public education.

Second, despite the much-hyped, neo-liberal promises of reducing teaching workloads, AI-driven solutions run counter to Bushido values and traditional Confucian work ethics in Japanese society. Such cultural proclivities call for unquestioning loyalty, long working hours, and ‘mandatory volunteerism’ (taking on extra ‘unpaid’ overtime duties overseeing extracurricular activities) [31,32]. The degree to which these cultural and financial tensions are unraveling makes for a unique and compelling study of the various social and ethical concerns of emotional AI in the Japanese educational system.

The prevailing narrative behind implementing AI in educational settings suggests it will enhance the effectiveness of learning by identifying children in need and define better methods of course delivery for the students. The end goal of this edtech plan is to shorten not just instruction time but also preparation time for students to enter the workforce. This neoliberal, techno-driven pedagogy is epitomized in the Japanese government's 2019 “AI Quest” initiative, launched by the Ministry of Economy, Trade and Industry (METI, 2022) which claims AI will aid in producing able bodies for the Japanese labor market. But, in truth, it neglects a larger set of problems such as systemic issues within the educational system, stigmas associated with the teaching profession and importantly, a future workforce threatened by a declining population. For example, a recent study found that over 70 % of junior high school teachers in Japan are overworked by more than 80 hours each month, which technically meets the government threshold for determining death by overwork (過労死―karoshi) [33].

Conversely, there are optimists who believe that the Japanese educational system can benefit from AI. In a comprehensive overview of the current adoption of educational AI tools in Japan, Yamada Seiji (2018) identifies emotional AI as an important heuristic for supporting the learning process and enhancing pedagogical effectiveness. Seiji cites the popularity of the foreign language app, *duolingo* in Japan [34]. He notes how the app's gamified features such as role-playing, achievement levels, award badges, and sonic effects generate exciting and fun learning experiences for Japanese users. Because rote learning is standard practice in the classrooms, Seiji argues that it is important to leverage Japanese children's penchant for smartphones and gamification, pointing out that over 28 million Japanese smartphone gamers are mainly elementary, junior high, and high school students. Problematically, he also notes that these users are predominantly male.

### 1.2 Educational AI technologies in Japanese education

AI startups specializing in edtech are becoming increasingly pervasive in Japan. For example, NEC, an electronics conglomerate in Japan, launched at the beginning of the global pandemic an AI tool that analyzes the emotions of participants in video conferences or Zoom classes [35]. Likewise, the Tokyo-based company, COMPASS, has created an AI-driven software that creates teaching materials configured to an individual student's learning level [36]. The AI-powered tool is reportedly used by about 500,000 people in more than 1800 elementary and junior high schools nationwide. COMPASS claims that since *Qubena* is integrated into the online learning system MEXCBT^1^ of the Ministry of Education, Culture, Sports, Science and Technology starting September 2022, the public's familiarity with adaptive learning systems will increase [37]. Similarly, *atama +*, is a tablet-based AI tool (now equipped with ChatGPT) that can assess students’ level of comprehension, mistakes, learning history, concentration, etc. in real time and proposes the shortest curriculum with the highest learning effect. The company states that its product can be found in over 3200 educational facilities across Japan and carries a proven track record of reducing students’ learning time by sixfold [36].

While interest in emotion-sensing edtech can be attributed to the pandemic and the public's heightened awareness of AI, it is important to note that digital learning in Japan is not something new. Since 1998, private businesses, such as the Uchida Yoko Institute for Education Research, have dedicated themselves to developing online learning and computer-based testing systems. Yet, government initiatives such as GIGA, only started after global studies revealed a below-average use of computers and the Internet among Japanese students [38]. These findings were followed by a surge in public spending on technological hardware improvements for educational institutions. The five-year infrastructure plan for ICT in education (2018–2022) allocated local governments with an annual budget of JPY180.5 billion (1.7 billion USD) [39], which means each elementary school receives an annual budget of approximately JPY6.22 million yen (almost 60,000 USD) [40].

Examining ‘Society 5.0’, the government's strategic blueprint for Japan's future with AI, in the education sector, Holroyd (2020) suggests that connecting education to the national priorities should not be an issue considering Japan's unitary state^2^ and the historical pattern of letting its strong central national government control the centralized education system [41]. Historically, however, Japan is plagued by a disconnect between political ambitions and bureaucrat pragmatism, centralized decision-making and regional reluctance at implementation. Even with the push of the COVID-19 pandemic toward remote learning, the rate of smart technologies adoption, including AI services, in Japanese schools is very slow. An interviewee, who is a high school teacher on the southern island of Kyushu stated: “There was a lot of discussions at our school about using more tablets and smartphones to facilitate remote learning during the pandemic. However, it never came into fruition” (Personal Communication, 2021). This statement is concurrent with a larger reality supported by existing literature suggesting a wide gap between government pro-technology messaging and actual practices which are dominated by factors at the local and cultural levels. Thus, it is important to investigate systematically positive reactions and concerns regarding emotional AI's integration in education and children's toys setting.

### 1.3 Research questions

With that said, this study is among the first to deploy the Technological Acceptance Model (TAM) and Moral Foundation Theory together to systematically investigate the perceived risks and rewards of emotional AI in education and children's toys setting. We focus on Japanese public perceives of the adoption and integration of emotional AI technologies in schools and toys, analyzing a national, representative dataset of 2000 Japanese respondents. In doing so, this study answers the following three research questions.

• RQ1: How do perceptions of emotional AI applications vary across demographics (sex, age income, educational qualifications)?
• RQ2: How do perceived utilities, self-rated AI knowledge, and concerns for fundamental values such as privacy, autonomy, safety, etc. correlate with the attitude toward emotional AI applications?
• RQ3: How do these correlations vary across context of applications, namely, from schools to children's toys?

This study examines emotional AI applications targeting the development of young minds in school settings and children's toys at home. Yet, given the growing popularity of emotional AI toys with children aged 1–12 years old, we suggest that they will also become a permanent fixture in pre-school, kindergarten primary school environments. Here, the combination of TAM and Moral Foundation Theory in the Three-Pronged Analytical Framework [42], which is further explicated in the Methods section, enables a systematic comparison of how Japanese people perceive the risks and rewards of emotion-sensing algorithms across two contexts: schools and toys. Doing so allows for a deepened understanding of how cultural notions of privacy, autonomy, and educational norms play a role in how AI technologies are integrated into Japanese society, which is where technological acceptance intersects with the realm of cultural and moral values. The goal of this article is to provide empirical insights and theoretical considerations for further research in the emerging field of socio-cultural analysis of AI and its impact on human-machine relations [42,43].

## 2 Materials and methods

### 2.1 Methods

**Fig. 1:**
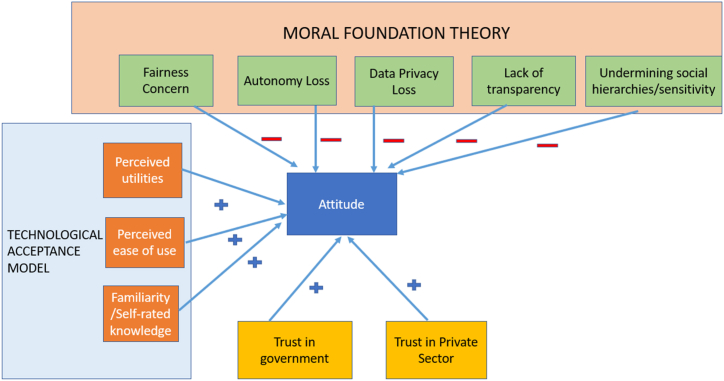
A visualization of how factors in TAM and Moral Foundation Theory are hypothesized to influence attitude toward emotional AI technologies.

This study leverages the two intuitions about technological acceptance, namely, the level of acceptance increases with perceived utilities and ease of use (formalized in the TAM—Technological Acceptance Model [44]), and acceptance decreases with the perceived violation of foundational moral norms and values (formalized in Moral Foundation Theory [45]). Thus, this study proposes that these two frameworks should be combined to better gauge technological acceptance in the age of emotional AI. We call this new framework the Three-pronged Approach and a full research paper explaining the details of this approach can be found in Ref. [42]. This new framework requires researchers to identify the relevant contexts (both context of use and cultural contexts), and then construct the studied variables by applying the TAM and Moral Foundation Theory to the contexts identified. Finally, the researchers need to apply appropriate statistical tools to understand the relationships among the variables. Fig. 1 presents a visualization of how various factors theorized in TAM and Moral Foundation Theory are predicted to influence attitude toward emotional AI technologies. In Table 1, all hypotheses that will be tested with quantitative analyses are presented.

**Table 1:** Hypotheses based on the theoretical frameworks and the literature.

No.	Hypotheses	Literature/Theories
1	H1: **Being male** is positively correlated with attitude toward emotional AI. While the opposite is true for **female**. (RQ1)	Empirical findings on attitude toward AI applications [[46], [47], [48]]/Sex differences in Moral Foundation Theory [[49], [50], [51]]
2	H2: **Female** respondents express more concerns about emotional AI's implications for moral harms such as privacy violation, autonomy loss, biased algorithms. (RQ1)	Sex differences in Moral Foundation Theory [49,50]
3	H3: **Perceived utilities** of emotional AI technologies positively correlate with attitude toward them (RQ2)	Predictions from Technological Acceptance Model [44,[52], [53], [54]]
4	H4: **Self-rated knowledge** with emotional AI technologies is positively correlated with attitude toward the emerging technologies.	Predictions from Technological Acceptance Model's [44,[52], [53], [54]]
5–1	H5-1: Concern about emotional AI's negative impacts on the moral value of **privacy** is negatively correlated with attitude toward emotional AI technologies.	Predictions from Moral Foundation Theory as adapted in the book How humans judge machines [[49], [50], [51]]
5–2	H5-2: Concern about emotional AI's negative impacts on **autonomy** is negatively correlated with attitude toward emotional AI technologies.	Predictions from Moral Foundation Theory as adapted in the book How humans judge machines [[49], [50], [51]]
5–3	H5-3: Concern about emotional AI's negative impacts on **fairness** is negatively correlated with attitude toward emotional AI technologies.	Predictions from Moral Foundation Theory as adapted in the book How humans judge machines [[49], [50], [51]]
6	H6: Concern about **accuracy of the technology** is negatively correlated with attitude toward emotional AI technologies.	Predictions from Moral Foundation Theory as adapted in the book How humans judge machines [[49], [50], [51]]
7	H7: **Transparency on data management** (how emotional data is managed, stored, processed) positively correlated with attitude toward emotional AI. The opposite is true when no transparency is provided.	Qualitative research results from various use cases including cars [10], toys [21,55], data management [56], education [20], smart homes, security [57]; workplace [12,58], etc.
8	H8: **Trust toward the government's ability to regulate** the technology is positively correlated with attitude toward emotional AI technologies.	Empirical findings from attitude toward AI/Robots and government effectiveness index [59]
9	H9: **Trust toward the private sector's ability to regulate** the technology is positively correlated with attitude toward emotional AI technologies.	Empirical findings from attitude toward AI/Robots and techno-social environment [59]
10	H10. (**The context sensitivity hypothesis**): Determinants of attitude toward emotional AI varied according to different contexts.	Qualitative research results from various use cases including cars [10], toys [21,55], data management [56], education [20], smart homes, security [57]; workplace [12,58], etc.

### 2.2 Materials

The empirical analysis is based on a national survey on Japanese population (N = 2000), which was distributed online during March 2022 through the market research company Cross Marketing. During March 2022, Cross Marketing distributed the online survey to 39,679 people and received 3301 responses. The company was contractually obliged to provide the final sample of 2000 responses with all age groups and sexes equally represented. A number of quality assurance methods has been applied to ensure the quality of the data, including the removal of all straight-line answers. For each use case, we ask the respondents to response to ten five-point Likert-scale statements (1 means strongly disagree to 5 means strongly agree), and each statement is based on a variable in either the TAM or the Moral Foundation Theory (See Table 2). In addition, five other socio-demographic information are also collected: age, sex, income level, educational qualifications, and regions (See Table 3).

**Table 2:** Variables, Items and Likert-scale items collected in “General Japanese citizens’ perception of emotional AI technologies,” a national survey on the Japanese population.

Emotional AI in schools Schools in some countries are employing companies to install cameras and artificial intelligence in classrooms to track students' facial expressions to try to work out their emotional states and attention levels. This aims to tailor teaching approaches by understanding if some students are struggling with class material or if other students need to be challenged more. It also aims to identify students’ attention levels, to help teachers to monitor and record in-class attention levels.		
Variables	**Statement** 1 (strongly disagree) to 5 (strongly agree)	**Scale**
AttitudeEAIschool	I would be comfortable with schools using emotion and attention monitoring in this way.	1 (strongly disagree) to 5 (strongly agree)
BiasConcern	I would be concerned that the emotion recognition software would not work consistently across children of different genders, ethnicities, ages, and disabilities. Some children could end up misclassified, and so get inappropriately tailored teaching or punishment.	1 (strongly disagree) to 5 (strongly agree)
DataMisuseConcern	I would be concerned about what happens to the emotional data about the child, and whether it might be used against the child in some way (now or in the future).	1 (strongly disagree) to 5 (strongly agree)
DystopianConcern	This sort of emotional monitoring would feel dystopian. Children could worry about being judged by machines on their facial expressions at school.	1 (strongly disagree) to 5 (strongly agree)
Knowledge	I have a basic understanding of the emotion-sensing technologies involved in such educational practices and their uses.	1 (strongly disagree) to 5 (strongly agree)
SafetyUtility	I consider this use of emotion-sensing AI systems improve the safety of the school.	1 (strongly disagree) to 5 (strongly agree)
AccuracyConcern	I am concern about the overall accuracy of such emotional AI systems.	1 (strongly disagree) to 5 (strongly agree)
TrustGov	I think the government will be capable of providing sufficient regulations for such uses of emotional AI technologies.	1 (strongly disagree) to 5 (strongly agree)
TrustPrivate	I trust companies to regulate themselves, ensuring that their technology will not result in racial, gender or age bias/discrimination and privacy harm.	1 (strongly disagree) to 5 (strongly agree)

Emotional AI Toys This question is about interactive toys for children up to 12 years old. Toymakers are interested in building toys with capabilities for basic conversations, meaning they can increasingly understand and derive meaning from children's speech. These toys would also try to interpret emotion in child speech, through tone of voice, so that the toy can respond appropriately by adapting play activities or trying to cheer them up if they are sad.		
Variables	**Items**	**Scale**
AttitudeEAIToys1	1. I would be comfortable with this as it sounds like fun. I wish I had toys like this when I was younger.s	1 (strongly disagree) to 5 (strongly agree)
UndueInfluence	2. I would have concerns about what the toy is saying to the child, how it is handling conversation with the child, and maybe even what it is advising the child to do or think.	1 (strongly disagree) to 5 (strongly agree)
DataManage Concern	3. I would have concerns about where the emotion data about conversations would go and who could access it e.g. advertisers trying to sell the child more toys.	1 (strongly disagree) to 5 (strongly agree)
OK AliveIlusion	4. I am comfortable with the idea that a young child might perceive the toy's artificial personality as something that is conscious or alive.	1 (strongly disagree) to 5 (strongly agree)
Privacy Concern	5. I consider this practice too much scrutiny of my child's emotions.	1 (strongly disagree) to 5 (strongly agree)
Knowledge	6. I have the basic understanding of emotion-sensing technologies involved in such toys.	1 (strongly disagree) to 5 (strongly agree)
Accuracy Concern	7. I am concerned about the overall accuracy of such emotional AI systems.	1 (strongly disagree) to 5 (strongly agree)
Bias Concern	8. I would be concerned that the emotion recognition software would not work consistently across children of different genders, ethnicities, ages, and disabilities.	1 (strongly disagree) to 5 (strongly agree)
TrustGov	9. I think the government will be capable of providing sufficient regulations for such uses of emotional AI technologies.	1 (strongly disagree) to 5 (strongly agree)
TrustPrivate	10. I trust companies to regulate themselves, ensuring that their technology will not result in racial, gender or age bias/discrimination and privacy harm.	1 (strongly disagree) to 5 (strongly agree)

**Table 3:** Socio-demographic factors from a national, representative survey on the Japanese population.

AGE AND GENDER		Frequency 2000	% 100.0
1	Male／20s	200	10.0
2	Male／30s	200	10.0
3	Male／/40s	200	10.0
4	Male／50s	200	10.0
5	Male／60s	200	10.0
6	Female／20s	200	10.0
7	Female／30s	200	10.0
8	Female／40s	200	10.0
9	Female／50s	200	10.0
10	Female／60s	200	10.0
EDUCATIONAL LEVEL		N = 2000	100 %
1	Middle School	42	2.1
2	High School	530	26.5
3	Colleges of technology (高等専門学校)	37	1.9
4	Vocational School (専門学校・専修学校)	235	11.8
5	Junior college (短期大学)	186	9.3
6	Bachelor's	852	42.6
7	Master's	90	4.5
8	PhD	19	1.0
9	Others	9	.5
INCOME LEVEL		N = 2000	100 %
1	Under 3,300,000 JPY	450	**22.5**
2	Between 3,300,000 and 9,000,000 JPY	865	43.3
3	Between 9,000,000 and 18,000,000 JPY	228	11.4
4	Over 18,000,000 JPY	43	2.2
5	Do want to answer	414	20.7

### 2.3 Ethical approval and informed consent

Before its distribution, the survey was screened and received an ethical Approval number 2020-07 from the Ritsumeikan Asia Pacific University's Research Compliance/Ethics Review Committee. Before answering the survey, the respondents were given a clear description that the description of the survey states that All information obtained from the survey are used to generate broad statistical understanding of social perceptions regarding emotional AI applications, the respondents remain anonymous, and identities such as residence address, birth date, names were not required, and they can choose to leave the survey at any point. Regarding informed consent, after reading the description, the respondents are informed that by choosing to answer the questions in the survey, their informed consent is given for the data generated from the answers being used for further studies.

## 3 Results

### 3.1 Emotional AI in Japanese schools

#### 3.1.1 Descriptive statistics and sex differences

The respondents are asked to respond to a series of Likert-scale questions (scale 1 to 5, 1 being strongly disagree, to 5 being strongly agree) on various aspects regarding the use of emotional AI in school. There are nine questions in total, each is pertaining to one social aspects of emotional AI in school setting; for example, attitude toward emotional AI, concern about biases in AI systems, concern about autonomy violation, concern about how the data is managed, utilities of the technology, concern about accuracy of the technology, etc. (A full list of question statements is provided in Table 2). The questions are designed based on the three-pronged approach, i.e. the survey questions are designed with consideration for context of technological application (e.g., in schools or in toys), cultural context, and suitable statistical method.

**Fig. 2:**
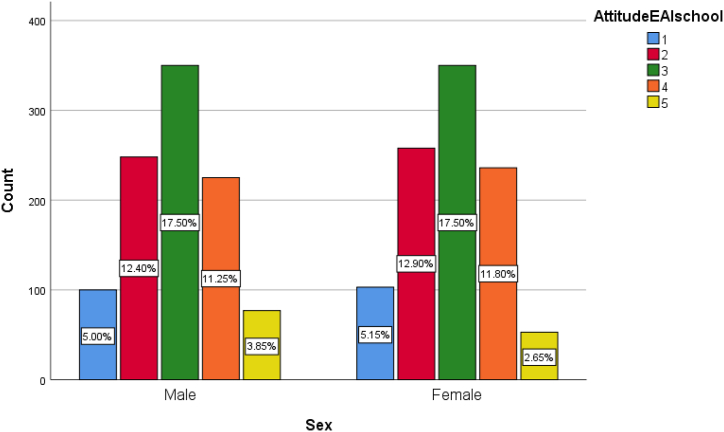
Distribution of attitude toward emotional AI in schools by sex. 1 means strongly disagree, 5 means strongly agree.

The descriptive statistics in Fig. 2 show that there are slightly more people who report feeling *negative* about emotional AI in schools (35.4 %) than those who report feeling *positive* (29.6 %). Meanwhile, 35 % of the respondents stay neutral on the topic. The mean score for the attitude toward emotional AI in schools is 2.9 (sd = 1.068), suggesting Japanese people are on average more negative about the use of emotional AI in schools. Table 4 present the descriptive statistics on sex differences regarding attitude toward emotional AI in schools.

**Table 4:** Sex differences regarding attitude toward and concerns about emotional AI in schools.

Sex		AttitudeEAI school	Bias Concern	DataMisuse Concern	Dystopian Concern
Male	Mean	2.93	3.49	3.41	3.39
	N	1000	1000	1000	1000
	Std. Deviation	1.085	.946	.969	.987
**Female**	**Mean**	2.88	3.67	3.58	3.56
	**N**	1000	1000	1000	1000
	**Std. Deviation**	1.051	.839	.855	.858
**Total**	**Mean**	2.90	3.58	3.49	3.47
	**N**	2000	2000	2000	2000
	**Std. Deviation**	1.068	.898	.917	.928

Sex		Knowledge	Safety Utility	Accuracy Concern	TrustGov	TrustPrivate
Male	Mean	2.99	3.09	3.46	2.83	2.88
	N	1000	1000	1000	1000	1000
	Std. Deviation	.969	.951	.951	1.004	.960
**Female**	**Mean**	2.95	3.07	3.57	2.83	2.93
	**N**	1000	1000	1000	1000	1000
	**Std. Deviation**	.875	.855	.865	.932	.903
**Total**	**Mean**	2.97	3.08	3.51	2.83	2.91
	**N**	2000	2000	2000	2000	2000
	**Std. Deviation**	.924	.904	.911	.968	.932
RANGE:	1 (Strongly disagree) to 5 (strongly agree)					

Running the Chi-square test, we find statistically significant sex differences among the following variables: the concern that EAI used in school to monitor emotion and attention can be biased against certain disadvantaged groups (BiasConcern, p < 0.001); the concern that emotional data of children collected by EAI might be used against them now or in the future (DataMisuseConcern, p < 0.001); the concern that EAI in school is dystopian as emotional expressions of children are constantly monitored (DystopianConcern, p < 0.001); self-rated knowledge about EAI use in school (Knowledge, p = 0.017); the recognition of increased safety due to EAI use in school (SafetyUtility, p = 0.003); the concern about accuracy of EAI systems (AccuracyConcern, p = 0.002). Meanwhile, there is no meaningful sex differences in the variables of trust in the government or the private sector's ability to regulate the technology (TrustGov and TrustPrivate) as well as the variable of attitude toward EAI use in school (AttitudeEAIschool).

Thus, we find female respondents are, on average, more concerned about the potential biases in EAI systems being used in school, the potential for data misuse, the dystopian feature of constantly monitoring children's emotions, and the potential for inaccurate reading of emotions compared to their male counterparts. Female respondents are also less positive about the increased safety utility in school as the result of using EAI systems and rate themselves as having less understanding of the technology.

#### 3.1.2 Regression analysis

i. Socio-demographic factors

Regarding the socio-demographic determinants of attitude toward EAI in school (Table 5), only age exhibits a statistically significant negative correlation with the attitude toward EAI in school (β_Age_ = - .129***).

ii. Utility, Values and Concerns

**Table 5:** Regression results for socio-demographic factors and attitude toward emotional AI in schools.

Model		Unstandardized Coefficients		Standardized Coefficients	t	Sig.
		B	Std. Error	Beta		
**1**	**(Constant)**	3.323	.130		25.560	.000
	**Age**	−.010	.002	−.129	−5.161	.000
	**Income**	.023	.039	.015	.596	.551
	**Education**	.001	.015	.001	.055	.956

a. Dependent Variable: AttitudeEAIschool; R square = .017.

This model explains 44.5 % of the variation in the data. As Table 6shows, positive correlates of attitude toward emotional AI in education include the recognition of increased safety as a consequence of using EAI in school (β_SafetyUtility_ = .356***); concerns for biases toward disadvantaged groups (β_BiasConcern_ = .065**); self-rated knowledge of the technology (β_Knowledge_ = .164***); having trust in the government's regulation (β_TrustGov_ = .091***); having trust in the private sector to regulate the technology (β_TrustPrivate_ = .17***). With safety utility being the strongest correlate, the surveyed population considers increased safety at school, which includes but is not limited to smart camera surveillance, intelligent tutoring systems, secured computer-based testing, and anxiety monitoring function, as a major advantage the technology will offer for education. Here, the results resonate with the TAM's predictions on perceived utility and perceived ease of use, as well as, the importance of the techno-social environment and government effectiveness in enhancing technological adoption [59] (see Table 7).

**Table 6:** Regression results for behavioral determinants of attitude toward emotional AI in schools.

Model		Unstandardized Coefficients		Standardized Coefficients	t	Sig.
		B	Std. Error	Beta		
**1**	**(Constant)**	.564	.111		5.060	.000
	**BiasConcern**	.077	.029	.065	2.643	.008
	**DataMisuseConcern**	−.025	.030	−.021	−.835	.404
	DystopianConcern	−.109	.030	−.094	−3.650	.000
	Knowledge	.190	.025	.164	7.643	.000
	SafetyUtility	.421	.026	.356	16.108	.000
	AccuracyConcern	−.051	.028	−.043	−1.830	.067
	TrustGov	.100	.026	.091	3.796	.000
	TrustPrivate	.195	.028	.170	6.926	.000

a. Dependent Variable: AttitudeEAIschool; R square = .445.

**Table 7:** Sex differences regarding attitude toward and concerns about emotional AI in toys.

Sex		AttitudeEAI Toys	UndueInfluence	DataManage Concern	OK AliveIlusion	TrustPrivate
Male	Mean	3.32	3.31	3.40	3.09	2.96
	N	1000	1000	1000	1000	1000
	Std. Deviation	1.019	.919	.939	.924	.959
**Female**	**Mean**	3.33	3.41	3.54	3.09	3.02
	**N**	1000	1000	1000	1000	1000
	**Std. Deviation**	1.021	.851	.896	.903	.851
**Total**	**Mean**	3.32	3.36	3.47	3.09	2.99
	**N**	2000	2000	2000	2000	2000
	**Std. Deviation**	1.020	.887	.921	.913	.907

Sex		Privacy Concern	Knowledge	Accuracy Concern	Bias Concern	TrustGov
Male	Mean	3.13	3.06	3.36	3.37	2.89
	N	1000	1000	1000	1000	1000
	Std. Deviation	.960	.898	.897	.897	.992
**Female**	**Mean**	3.24	3.05	3.45	3.47	2.91
	**N**	1000	1000	1000	1000	1000
	**Std. Deviation**	.888	.866	.850	.817	.881
**Total**	**Mean**	3.19	3.05	3.40	3.42	2.90
	**N**	2000	2000	2000	2000	2000
	**Std. Deviation**	.926	.882	.875	.859	.938

**Table 8:** Correlations of socio-demographic factors and attitude toward emotional AI toys.

Model		Unstandardized Coefficients		Standardized Coefficients	t	Sig.
		B	Std. Error	Beta		
**1**	**(Constant)**	3.933	.105		37.477	.000
	**Age**	−.010	.002	−.133	−5.984	.000
	**Income**	−.007	.016	−.009	−.413	.680
	**Education**	−.034	.012	−.062	−2.793	.005

a. Dependent Variable: AttitudeEAItoys; R square = .02.

A notable paradoxical result is the positive correlation between the bias concern and the attitude toward EAI in school. In other words, even though this study finds Japanese participants to be concerned about the biased treatment of disadvantaged groups, their attitude toward emotional AI in school remains positive. This result implies that people are willing to accept the technology regardless of its latent biases. This positive correlation perhaps speaks to the long-standing cultural belief in an egalitarian and homogenous Japanese society regardless of existing contradictions [60].

In terms of negative correlates, people who agree that affect-sensing algorithmic tools constantly monitoring children's emotions are too dystopian are more likely to disagree that such use of the technology will be beneficial for society (β_DystopianConcern_ = − .094***). The result aligns with the Moral Foundation Theory assumption that a violation of privacy via the empathic surveillance should increase unease toward the technology. This confirms Kucirkova et al. [61]'s findings that privacy concern is among the key considerations of Japanese parents and teachers regarding personalized digital learning devices. Kucirkova et al. [61] also highlight the risks posed to children's safety by the disclosure of personal information and the difficulty of ensuring personal data security in the Japanese educational setting. More importantly, the result stresses the need for transparency, in terms of data collection, data management, data sharing and data retention. Clearly, in this context, it is useful to consider the data minimization principle expressed in Article 5(1) of the GDPR, which limits the collecting and processing data only toward necessary ends. Yet, according to legal analyses, the principle is open to interpretations and the facts that data are shared among multiple parties makes it harder to define what constitutes the minimal necessary [62].

Interestingly, concerns for data misuse (i.e., answer to the question “I would be concerned about what happens to the emotional data about the child, and whether it might be used against the child in some way (now or in the future).”) and concerns about accuracy of the technology have no statistically significant relationship with the attitude toward EAI in school. This result somewhat contradicts Kucirkova et al. [61]'s finding that worries of data misuse and how the technology might influence students being the key concerns of Japanese teachers.

In the context of existing literature, the findings from the regression analysis highlight the ambivalent attitude toward affect tools in the Japanese classroom. The authors of these studies find while teachers and parents welcome the benefits of new technologies’ (i.e. personalized learning), they also feel that the technology must be closely monitored by responsible adults [61].

Given the fact that there is still an ongoing debate on the nature of human emotions, whether they are wired in our physical biology or socially constructed, it is clear that we need to be cautious with the use of EAI in school settings. Before considering EAI as a quick fix solution tech-solution for students’ low motivation and performance, more wide-ranging cultural, structural, and systemic factors need to be considered. Japanese culture is well-known for its long-standing affinity for intelligent machines (from Astro-Boy to Doraemon) but at the same time, it also has a reputation as a culture which is reluctant to embrace institutional change. Simply augmenting the Japanese public educational system with EAI edtech does not necessarily mean it will alleviate the cultural demands on work ethics placed on teachers, nor will it liberalize traditional practices and methods of learning. The importance of addressing these structural issues before thinking of using EAI to monitor and modify students’ behaviors, concurs with observations and arguments by a growing number of scholars [20].

### 3.2 Emotional AI in children's toys

Human-toy relationships have played a formative role in a child's social and emotional development, often augmenting a parent's tutoring role in this evolution. Take for example, the gendered nature of baby dolls, the child-rearing expectations it places on young females. Or conversely, the intensely masculine and militarized GI Joe action figurine, its interpellative effect on young male subjectivities. Given the growing pervasiveness of intelligent machines in society, it is not surprising that toy manufacturers are embedding AI into new product lines of children's playthings. A good illustration can be found in the marketing claims of the EAI toy makers such as Embodied whose latest smart toy ‘Moxy’ is said to foster greater levels of intimacy than normal toys which in turn enhances a child's social and emotional development. However, a growing number of critics warn that AI-driven toys may, in fact, open children to a myriad of harms such as hacking or data theft by malicious actors [63], algorithmic bias or discrimination, eroding a parent's ‘duty of care’ [27] and forcing attitudinal conformity upon children [28].

#### 3.2.1 Descriptive statistics and sex differences

Below is the explanation to survey respondents about the use of EAI toys. The respondents were asked to give their reply on the scale of 1 (strongly disagree) to 5 (strongly agree) to various statements regarding the utilities and concerns implicated in the use of emotional AI in children's toys.

“This question is about interactive toys for children up to 12 years old. Toymakers are interested in building toys with capabilities for basic conversations, meaning they can increasingly understand and derive meaning from children’s speech. These toys would also try to interpret emotion in child speech, through tone of voice, so that the toy can respond appropriately by adapting play activities or trying to cheer them up if they are sad.”

There were ten Likert-scale questions in total, each is pertaining to one social aspects of EAI in children's toys; for example, attitude toward emotion-sensing, concern about biases in AI systems, concern about autonomy loss, concern about how data are managed, utilities of the toys, concern about accuracy of the technology, etc. (A full list of question statements is provided in Table 2).

**Fig. 3:**
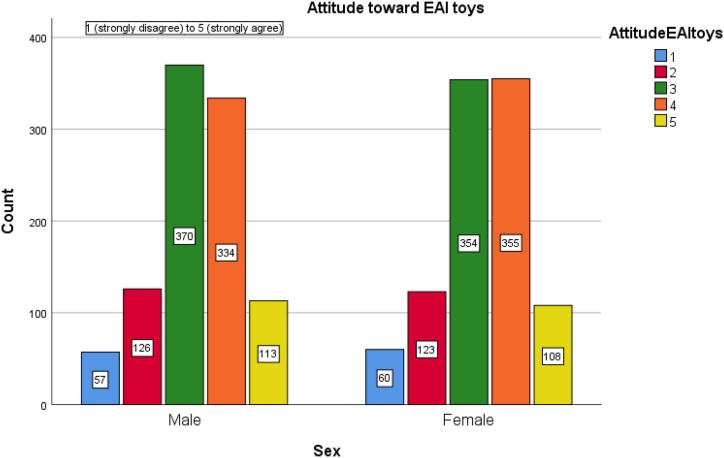
Distribution of attitude toward emotional AI in children's toys by sex. 1 means strongly disagree, 5 means strongly agree.

Fig. 3 shows the distribution of answers regarding the attitude toward emotional AI in toys. Compared to the school case, emotional AI in toys receives more acceptance from the Japanese population. Overall, 36.2 % report feeling neutral about such toys, while 34.5 % and 11.1 % report feeling somewhat positive and very positive, respectively, regarding emotional AI in toys. Thus, about 46 % report being positive and accepting of the emerging technology, while only 17 % report feeling negative about the technology. It is worth noting that only 5.9 % report a strong disagreement regarding the technology. The willingness to embrace this technology in children's toys is in line with [61] aforementioned [61]. note albeit likelihood of children interacting with such technologies in the future is inevitable there is a need to ensure children's agency in using these smart, personalized toys.

Running the Chi-square test, we find statistically meaningful differences between the sexes in the following variables: the concern about undue influence of the EAI toys on children (UndueInfluence, p = 0.026); the concern about how emotional data of the children are managed (DataManagementConcern, p < 0.001); the concern about loss of privacy or too much monitoring of children emotions (PrivacyConcern, p = 0.01); the concern about overall accuracy of the technology (AccuracyConcern, p = 0.039); the concern about social biases embedded in emotional AI toys (BiasConcern, p = 0.011); the trust in government's regulation (TrustGov, p = 0.006); the trust in the private sector's regulation (TrustPrivate, p = 0.003).

Thus, we find that women express more privacy worries, more accuracy and bias concern, worry more about the interaction of the EAI toys with the children. These heightened fears of Japanese women align with the Moral Foundation Theory that posits women care more about the moral dimensions of Harm, Fairness, and Purity than men [49]. This is similar to the finding by Kucirkova and Toda [61], in which the teachers, primarily female, are more concerned than their male counterparts about the teacher-children relationships if a smart teddy bear can hold AI-powered conversations with the children. The reasons for this worry are attributed to the teachers’ desire to cultivate agency and autonomy in children as well as the deep-seated fear of personal data breaches. Overall, the literature on digitalized, smart, internet-connected toys is more focused on the issues of data privacy and human interactions than the more fine-tuned aspects of technological accuracy and inherent social biases [55,64,65].

#### 3.2.2 Regression analysis

iii. Socio-demographic factors

Running a regression analysis on socio-demographic factors and attitude toward emotional AI toys, we find that both age and education are negative correlates of the dependent variable (β_Age_ = - .133***; β_Education_ = −.062 **). Here, as elderly people tend to reject new emerging technologies, it is expected that age would negatively correlate with attitude toward emotional AI in toys [7]. It is unexpected that education is a negative correlate since the literature indicated that people with higher educational qualifications tend to view new, emerging technologies such as AI or robots more favorably. This result might be due to the subject being children's toys. There might be uncomfortable feelings among more educated participants regarding how the EAI toys might interact with or influence children.

Indeed, it is likely that the more educated the participants, the more they worry about the impact of technology on children's development. In fact, a growing body of psychological research indicates that compared with previous generations, a higher correlation exists between Gen Z ‘s technological dependency and mental health issues such as loneliness, depression, and anxiety [66], higher levels of individualism in learning and teamwork [67], as well as higher levels of reliance on digital devices for interpersonal communication [68].

iv. Values and concerns

This model explains 38 % of the variation in the data, which is quite low compared to other studies that use the extended TAM model [69,70]. Here, this result highlights the novelty of emotional AI in toys. Positive correlates of attitude toward emotional AI in toys include being OK with a child having an illusion that the toys might be alive and having a personality (β_OKAliveIllusion_ = .372***); concerns regarding the management of emotional data collected by the toys (β_DataManagementConcern_ = .118***); concerns for biases toward disadvantaged groups (β_BiasConcern_ = .067**); self-rated knowledge of the technology (β_Knowledge_ = .087***); having trust in the government's regulation (β_TrustGov_ = .091***); having trust in the private sectors to regulate the technology (β_TrustPrivate_ = .149***).

The strongest positive correlate is between being OK with the illusion of a toy's anthropomorphism, which suggests the surveyed population considers the feeling that the toy has a personality, and its human-like qualities are a major advantage when choosing whether to buy an emotional AI toy. However, the second strongest correlation is a *negative* one, which is between privacy concern and attitude toward emotional AI in toys. Here, people who agree that emotional AI toys that constantly monitors children's emotions is too intrusive are more likely to disagree that they want emotion-recognition in toys (β_PrivacyConcern_ = − .199***). Thus, there is tug of war between the utility of the toys presenting an illusion of being alive with privacy concerns.

It is interesting and seemingly paradoxical that concern about data management and concern about embedded biases are positive correlates with attitude toward emotional AI toys while accuracy concern is not a statistically significant predictor. Similar to emotional AI use in schools, the respondents’ lack of concern about algorithmic biases embedded in the toys might also be due to the homogenous nature of Japanese society. Moreover, the lack of concern for embedded bias may also come as a result of the manufacturers’ marketing claims that present the toys as products of innovative science [71]. Here, these same two reasons may also account for the absence of privacy concern by the respondents for outside parties who might access the emotional data of their children. These findings align to our initial worries regarding the lack of emotional AI toys and the importance of Japanese parents to have a basic understanding of how the technology operates to mitigate harms and risks to their children.

Concurringly, another interesting result is respondents’ concerns for data misuse and concerns for accuracy of the emotional AI toys have no statistically significant relationship. When we take a normative stance, this result is somewhat worrying, especially when we consider the aforementioned ‘generational unfairness” facing children. Since adults make toy purchasing decisions for children, our analysis shows concerns about data misuse and accuracy do not figure into their attitude toward emotional AI in toys. The results support the concern that McStay and Rosner (2021) raise regarding parents’ susceptibility and naivety since most parents lack literacy on how these toys collect, process, archive and share and archive user data. Consequently, the rise of emotional AI toys and their increasing presence in the home require an urgent need to educate parents and responsible parties to better understand the opaque workings of this new technology as well as its social and ethical implications to protect vulnerable children from any possible negative outcomes.

## 4 Discussion

### 4.1 Policy implications

This study, following the Three-Pronged Analytical Framework, brings together the Technological Acceptance Model and the Moral Foundation Theory to investigate the awareness of moral risks in the acceptance of emotional AI technologies in two contexts of young minds’ development: emotional AI in schools and in toys. Studying a sample of the Japanese population, we identify several results that potentially have relevant policy implications.

#### 4.1.1 Diverse representation in policymaking about social integration of AI

The empirical results highlight the importance of adequately representing diverse social interests in decision-making bodies. Concurring with the empirical literature on the sex differences in moral concerns, Japanese female respondents express more concerns regarding potential moral harms implicated in the unchecked use of the technology, e.g., biased algorithms, privacy violation, autonomy loss, etc. (See Table 3). These findings’ implications are twofold. First, it brings into sharp relief the necessity of women's representation in policy or regulatory bodies, especially, when it comes to sensitive matters such as the development of young minds. Second, it highlights the worrying lack of women representation in STEM education and decision-making bodies in Japan and worldwide [72].

#### 4.1.2 The necessity to equip parents and teachers with knowledge about emerging technologies

Since there have been well-established empirical results of the detrimental effects of smartphones and social media on the Gen Z population [68,73], future applications of emotional AI may help schoolteachers and parents to identify potentially harmful emotional and affective states in children. Conversely, by interaction with these smart toys, they may exacerbate or trigger negative behavioral and psychological tendencies in at-risk children. Given the malleability of young brains, this is worrying. Moreover, since this study shows there are significant percentages of Japanese respondents who demonstrate a lack of care on the accuracy of the technology as well as how the data are managed, there is a need for greater public outreach initiatives to educate both teachers and parents on the potential risks of emotional AI technologies.

This study also points out that the concern about data management issues for emotional AI in schools and toys does not factor into the overall attitude toward the technology. As argued previously, this might be a reflection of the nascent nature of emotional AI. Yet, this result calls attention to the importance of higher social demands put on transparency in how data are collected and processed when using emotional AI software products in schools or when interacting with smart toys. Parents and schoolteachers must be equipped with a basic understanding of how the technologies work as well as exercising caution when using the smart technologies with impressionable youth. It is also very important to respect the data minimization principle that has been put into official documents by Europe's General Data Protection Regulation and UNESCO's International Declaration on Human Genetic Data ([20]. Before regulatory frameworks are established, policymakers would be well-advised to include public discussion on the social norms and cultural values that need to be considered to ensure that emotional AI edtech serves the best interests of Japanese youth (see Table 9).

#### 4.1.3 Cross-cultural differences in norms and values regarding emotional AI's social integration

Third, regarding fundamental values and norms, this study shows that Japanese people are worried about the privacy implications of emotional AI in both cases. We find a negative significant correlation between concern about privacy violation and attitude toward emotional AI in both schools and toys. However, there are two differences when it comes to the values of autonomy and biases. There is a significant negative correlation between concern about autonomy and attitude toward emotional AI in the case of schools, but not in the smart toys case. And regarding the fairness dimension, concern about fairness has not significant relation to the attitude toward emotional AI, which hints at the prevailing perception of Japan as a homogenous society among the respondents. These culturally entangled results must be further studied in the future and at least be discussed among policymakers to create AI policies that suit their culture and social norms. Finally, Table 10 summarizes the decision made for each hypothesis based on the quantitative analysis.

**Table 9:** Correlations of behavioral factors and attitude toward emotional AI toys.

Model		Unstandardized Coefficients		Standardized Coefficients	t	
		B	Std. Error	Beta		Significance
**1**	**(Constant)**	1.117	.115		9.748	.000
	**UndueInfluence**	−.020	.028	−.018	−.730	.466
	**DataManagementConcern**	.131	.027	.118	4.789	.000
	**OKAliveIlusion**	.415	.025	.372	16.779	.000
	**PrivacyConcern**	−.219	.025	−.199	−8.749	.000
	**Knowledge**	.100	.025	.087	3.962	.000
	**AccuracyConcern**	−.039	.030	−.034	−1.290	.197
	**BiasConcern**	.080	.029	.067	2.807	.005
	**TrustGov**	.099	.028	.091	3.560	.000
	**TrustPrivate**	.167	.030	.149	5.571	.000

a. Dependent Variable: AttitudeEAItoys; R square = .38.

**Table 10:** A summary of decisions on the hypotheses examined in this study.

No.	Hypotheses	Decision	Research questions
1	H1: **Being male** is positively correlated with attitude toward emotional AI. While the opposite is true for **female**.	*Rejected:* Toys; Schools;	RQ1
2	H2: **Female** express more concerns about emotional AI's implications for moral harms such as privacy violation, autonomy loss, biased algorithms.	Supported in all cases.	RQ1/RQ2
3	H5: **Perceived utilities** of emotional AI technologies positively correlate with attitude toward them	Supported in all cases.	RQ2
4	H6: **Self-rated knowledge** with emotional AI technologies is positively correlated with attitude toward the emerging technologies.	Supported in all cases.	RQ2
5–1	H5-1: Concern about emotional AI's negative impacts on the moral value of **privacy** is negatively correlated with attitude toward emotional AI technologies.	*Supported:* Education; Toys;	RQ2
5–2	H5-2: Concern about emotional AI's negative impacts on **autonomy** is negatively correlated with attitude toward emotional AI technologies.	Rejected: Toys Supported: Schools.	RQ2
5–3	H5-3: Concern about emotional AI's negative impacts on **fairness** is negatively correlated with attitude toward emotional AI technologies.	*Rejected:* Education; Toys	RQ2
6	H6: Concern about **accuracy of the technology** is negatively correlated with attitude toward emotional AI technologies.	*Rejected*: Education; Toys	RQ2
7	H7: **Transparency on data management** (how emotional data is managed, stored, processed) positively correlated with attitude toward emotional AI. The opposite is true when no transparency is provided.	Rejected: Toys; education	RQ2
8	H8: **Trust toward the government's ability to regulate** the technology is positively correlated with attitude toward emotional AI technologies.	Supported in all cases	RQ2
9	H9: **Trust toward the private sector's ability to regulate** the technology is positively correlated with attitude toward emotional AI technologies.	Supported in all cases	RQ2
10	H10 (**The context sensitivity hypothesis**): Determinants of attitude toward emotional AI varied in according to different contexts.	Supported per the decisions shown above.	RQ3
A1	Income is positively correlated with the attitude toward emotional AI.	Rejected: Toys, Education	RQ1
A2	Age is negatively correlated with attitude toward emotional AI.	Supported in all cases.	RQ1
A3	Higher educational qualification positively correlated with attitude toward emotional AI.	Rejected: Toys; Education	RQ1

Note: A1, A2, A3 are additional hypotheses.

### 4.2 Limitations

This study is limited in several ways. First, due to its exclusive focus on Japan, caution must be applied when generalizing the results in a wider international context. This is important even though the survey was intentionally designed to be culturally sensitive albeit drawing insights from ‘universal’ theories of TAM and Moral Foundation Theory. This leads to the second limitation: due to constraints set by the online format provided by the market research company, not all values that might be important in the Japanese educational and child-rearing culture are accounted for. Thus, this is an area where future studies can address by synthesizing the literature on Japanese values and philosophies in education and children's development to design a more comprehensive survey. Nonetheless, given the wide range of values, concerns, and utilities examined and the concrete basis in widely accepted theories of human judgment toward technologies, this study has provided a first contribution to the quantitative analysis of cultural factors in the human judgment of technologies.

Second, it must be acknowledged that this study's survey format with one Likert-based question per variable such as autonomy, fairness, accuracy etc. might not capture the intricate thoughts a respondent has about these variables. Clearly, values such as autonomy, fairness, accuracy, etc., are rather complex and culturally sensitive variables as they can be revealed through various dimensions. This is where future studies can improve upon the survey design of this study by adding more items to each variable and using statistical tools such as factor analysis to create the variables. The issue of scale development is indeed an area of interest in survey-based methods for studying human-AI interactions [74,75]. It is worth noting that this very issue is also discussed in the paper on the Three-Pronged Analytical Framework for studying the moral risk of technological acceptance [42], which this study is based on.

Third, it must be acknowledged that the analysis is built upon an assumption that attitude toward smart machines follows a linear function, in which, acceptance is higher with its utilities and lower when the technology implicates a loss in social norms and values. This might not be the case given the numerous features of complexity (e.g., non-linearity, networked nature, and feedback loop [76]) laden in the emerging AI-human interactions. Specifically, emotional AI and other AI technologies such as AI chatbots [77] are increasingly designed to operate in the background of private and public spaces to modify our behaviors in the preconceived ‘appropriate ways’, hence, gauging technological acceptance of emotional AI might be more complex than purported by Davis's TAM [44] and Hidalgo et al. [51]'s Moral Space in *How humans judge machines*.

### 4.3 Future research directions

Our results indicate a certain alignment with results identified by Gkinko and Elbanna (2023) [77] that cognitive trust in AI chatbots (i.e., trust initially formed on the sense of familiarity and performance) are complemented by emotional and organizational trust. Gkinko and Elbanna (2023) show as users become emotionally more committed to the development and usage of AI chatbots, their sense of trust (in the chatbots) advanced, and emotional and organizational trust, once formed, are recalcitrant as they are hard to deteriorate *even when performance is poor*. This trust-forming process is analogous to the value-filtering process explored in mindsponge theory [78,79].

Here, arguably, we are better off thinking about our relationship with this new technology as a *value-filtering process*. In other words, future studies should consider systematic differences generated by cultural mindsets, an important factor in the Three-Pronged approach, by exploring the information filtering mechanism postulated by Vuong and Napier [79]'s *mindsponge framework* as a model of technological acceptance. Unlike TAM, the mindsponge framework considers the cost-and-benefit evaluation, i.e., the perceived usefulness and perceived ease of use in TAM, as not as the overriding factors in the filtering mechanism of the mind for new input. The ease of use as well as usefulness, in the mindsponge framework, acts as trust evaluators of the filtering process, e.g., higher perceived usefulness or higher ease of use help increase the trust in a technology, but they are not the overriding factors in determining its acceptance as the traditional TAMs suggest. Whether the mind of a user rejects or accepts an input is also contingent on auxiliary factors such as an individual's ability to creatively adapt new inputs to their specific circumstances but also how individual core values and external settings (cultural and political) reinforce or diminish the uses of such inputs [12,80]. Rather than adding new variables to the traditional TAM in a linear and somewhat arbitrary way, the mindsponge framework [78] combined with the Bayesian multi-level modeling [81,82] can offer a more systematic, hierarchical way of extending the TAM by differentiating between variables that come from an individual's core mindset, and the external cultural, ideological setting.

Thus, the mindsponge-based technological acceptance model can open new fertile grounds for future research, and below are a few suggestions for future studies to consider. We can incorporate various factors from the mindsponge model of information filtering to supplement the TAM and Moral Foundation Theory. These factors include personal core values (i.e., level of openness to experiences, level of creativity, level of religiosity); environmental factors of culture (i.e., regions of home country), and politics (i.e., political regime of the home country) [12,83]. In terms of modeling techniques, these factors form a varying intercept for a Bayesian network model or can be used in structural equation modeling as latent variables [84].

## 5 Conclusion

This article explores Japanese perceptions of the ethical implications of emotional AI in the context of educating and developing young learners in schools and with smart toys. In both cases, more respondents expressed a positive attitude toward such use of the technology than those two expressed negative feelings. We also find that older people are less receptive to the technology, with age being a negative correlate of attitude toward emotional AI in both cases.

In terms of sex differences, aligning with predictions from the Moral Foundation Theory, we find that women express more concerns about privacy, accuracy, and bias regarding emotional AI use in schools and toys. As for the regression analysis to understand how social and ethical perceptions of emotional AI influence acceptance of the technology, we find that self-rated knowledge and perceived utilities of the emotional AI (i.e., improving safety at school or making toys more interactive) are positive correlates of its acceptance. These findings agree with the predictions of the TAM. We also find that in both cases, trust in the regulation of the government and trust in the private sector positively correlate with the acceptance of the technology.

Importantly, we find two seemingly paradoxical results. The first is a positive statistically significant correlation between bias concern and attitude toward emotional AI in school. The second is about emotional AI toys: the concern about data management and the concern about embedded biases are positive correlates with attitude toward emotional AI toys. We can interpret the results as people accepting the technology even when they acknowledge its shortcomings. Moreover, the results might also reflect a cultural attitude regarding values such as privacy and homogeneity in Japan. These results would necessitate dialogue between policy-makers and researchers when coming up with culturally sensitive approaches to integrate AI into society.

## Data availability statement

The data associated with this manuscript can be accessed upon reasonable request.

## CRediT authorship contribution statement

**Manh-Tung Ho:** Writing – review & editing, Writing – original draft, Visualization, Software, Methodology, Formal analysis, Data curation, Conceptualization. **Peter Mantello:** Writing – review & editing, Writing – original draft, Resources, Project administration, Funding acquisition, Data curation, Conceptualization. **Quan-Hoang Vuong:** Writing – review & editing, Visualization, Validation, Methodology, Formal analysis.

## Declaration of competing interest

The authors declare that they have no known competing financial interests or personal relationships that could have appeared to influence the work reported in this paper.

## Footnotes

1 MEXCBT is the combination of MEXT (Ministry of Education, Culture, Sports, Science and Technology) and CBT (Computer Based Testing). MEXCBT, whose first prototype was introduced in 2020 and became official in November 2021, is envisioned as an online test bank that students, with one terminal per user, can use them to study. See https://g-apps.jp/ict-education/about-mexcbt-and-e-learning-portal/.

2 Japan has been led by the Liberal Democratic Party since the postwar constitution was made with only two brief windows of less than a year held by the main opposition party.
